# Epidemiology of Chikungunya Virus in Bahia, Brazil, 2014-2015

**DOI:** 10.1371/currents.outbreaks.c97507e3e48efb946401755d468c28b2

**Published:** 2016-02-01

**Authors:** Nuno Rodrigues Faria, José Lourenço, Erenilde Marques de Cerqueira, Maricélia Maia de Lima, Oliver Pybus, Luiz Carlos Junior Alcantara

**Affiliations:** Department of Zoology, University of Oxford, Oxford, United Kingdom; Department of Zoology, University of Oxford, Oxford, United Kingdom; Centre of Post-Graduation in Collective Health, Department of Health, Universidade Estadual de Feira de Santana, Feira de Santana, Bahia, Brazil; Centre of Post-Graduation in Collective Health, Department of Health, Universidade Estadual de Feira de Santana, Feira de Santana, Bahia, Brazil; Department of Zoology, University of Oxford, Oxford, United Kingdom; FIOCRUZ, Laboratory of Haematology, Genetics and Computational Biology, Salvador, Bahia, Brazil

**Keywords:** Chikungunya, disease outbreak, Epidemiology and surveillance, Virus

## Abstract

Chikungunya is an emerging arbovirus that is characterized into four lineages. One of these, the Asian genotype, has spread rapidly in the Americas after its introduction in the Saint Martin island in October 2013. Unexpectedly, a new lineage, the East-Central-South African genotype, was introduced from Angola in the end of May 2014 in Feira de Santana (FSA), the second largest city in Bahia state, Brazil, where over 5,500 cases have now been reported. Number weekly cases of clinically confirmed CHIKV in FSA were analysed alongside with urban district of residence of CHIKV cases reported between June 2014 and October collected from the municipality’s surveillance network. The number of cases per week from June 2014 until September 2015 reveals two distinct transmission waves. The first wave ignited in June and transmission ceased by December 2014. However, a second transmission wave started in January and peaked in May 2015, 8 months after the first wave peak, and this time in phase with Dengue virus and Zika virus transmission, which ceased when minimum temperature dropped to approximately 15°C. We find that shorter travelling times from the district where the outbreak first emerged to other urban districts of FSA were strongly associated with incidence in each district in 2014 (R^2^).

## Article

The Chikungunya virus (CHIKV) is an alphavirus that belongs to the Semliki Forest Virus antigenic complex^1^. Local outbreaks of CHIKV-like disease have been documented since the eighteenth century^2^ and the virus was discovered in 1952 in Tanzania^3^ . Over the last 50 years, CHIKV has spread beyond its African heartlands and caused explosive outbreaks comprising millions of cases in Indian Ocean islands and Asia.^4^The virus is mosquito-borne and transmission is associated with the vector species *Aedes albopictus* and *Ae. aegypti*, which are responsible for transmission cycles in urban and peri-urban environments^5^. Clinical symptoms include polyarthralgia, rash, high fever and severe headaches^6^ and outbreaks are often characterized by a rapid spread and high morbidity, resulting in losses in productivity^7^. To date, four distinct CHIKV genotypes have been identified. Two genotypes - the East-Central-South African (ECSA) and the West African genotypes - occupy a basal position in the phylogeny of CHIKV^8^ and are mostly enzootic in Africa. Of the remaining two genotypes, the Asian genotype is predominant in Southeast Asia, whilst the more recent Indian Ocean lineage spread from the Comoros islands in 2004 and caused a large outbreak in India and Southeast Asia in 2005-2008.

In October 2013, the Asian genotype was first reported in the Americas, in the island of Saint Martin in the Caribbean^9^. By the end of December 2015, nearly 1 million cases had been notified in the Americas, resulting in 71 deaths, and autochthonous transmission has been reported in more than 50 territories^10^. In September 2014 the Brazilian Ministry of Health confirmed autochthonous transmission of CHIKV in the Amapá federal state, which borders French Guiana in northern Brazil, and in Feira de Santana (FSA), a well-connected municipality and the second largest city (617,528 inhabitants in 2015, www.ibge.gov.br) of Bahia federal state (BA, northeast of Brazil, where both *Aedes* vectors circulate^11^. Genome sequencing revealed that the Asian genotype was circulating in the north of the country. However, 3 genomes sampled from FSA revealed that a distinct lineage, the ECSA genotype, had entered the Americas for the first time^12^. The ECSA genotype in FSA is closely related to CHIKV strains circulating in Angola. Despite sparse data on CHIKV in Angola, there are recent reports of CHIKV cases exported from Angola to other locations^13^. Angola is climatically suitable for *Aedes* spp. mosquitoes^11^^,^ 14 and CHIKV was previously isolated from *Aedes aegypti* in Luanda^15^. As a result of the two independent introductions of distinct CHIKV genotypes into Brazil, epidemiological analysis suggests that ~94% of the Brazilian population is at risk of CHIKV infection^12^. In 2015, the Brazilian Ministry of Health has notified a total of 20,661 CHIKV suspected cases – 7,823 (38%) of these have been confirmed with clinical (35%) and laboratory (3%) confirmation^16^ across 84 cities in Brazil - of which Feira de Santana has notified by far the greatest number of cases (4,088 only in 2015). It is likely that these numbers represent only a fraction of the individuals infected because CHIKV cases based on clinical diagnosis may be mistaken for dengue virus (DENV) or Zika virus (ZIKV) infection. DENV is hyperendemic in the country, with serotypes 1 to 3 being re-introduced every 7 to 10 years from other South American countries and the Caribbean^17^^,^ 18. Also, autochthonous transmission of ZIKV has recently been reported in several Brazilian federal states^16^, and although the origins of the virus remain less understood^19^, recent surveys indicate autochthonous transmission of ZIKV in 19 out of 27 federal states in Brazil.

The Brazilian surveillance health system (Sistema Nacional de Notificação de Agravos, www.saude.gov.br/sinanweb) defines a CHIKV suspected/notified case as an individual presenting with a sudden fever (>38.5 C) and arthralgia or intense acute arthritis that cannot be explained by other conditions, with the patient being either a resident or having visited endemic or epidemic areas two weeks prior to the onset of symptoms. A CHIKV confirmed case involves either epidemiological or laboratory confirmation through IgM serology, virus isolation, RT-PCR, or ELISA. In FSA, following the first RT-PCR and ELISA confirmed CHIKV cases in June 2014, CHIKV incidence was determined by epidemiological criteria on the basis of severe polyarthralgia or on the chronic evolution of symptoms, which enabled the municipal epidemiological surveillance teams to distinguish CHIKV from DENV and ZIKV infection.

We analysed temporal changes in CHIKV incidence among 21 urban districts of FSA between 1st June 2014 and 1st September 2015^20^. The data show evidence of a shift from a phase of epidemic establishment (June 2014 to December 2014) towards a pattern of transmission behaviour that matches well the annual incidence of DENV (**Fig. 1a**). Epidemiological surveys indicate that the ECSA index case visited the local polyclinic after arriving in the George Américo (GA) urban district in FSA on the 29th May 2014. The individual presented high fever and polyarthralgia and tested negative for DENV and malaria. Several family members and neighbours of the index case presented CHIKV-like symptoms in June 2014. As expected for a newly-emergent epidemic, we find that travelling time from the inferred epidemic origin (GA) to other locations, measured as the driving time in minutes from GA to the centre of each urban district, is inversely associated with incidence during the earliest stages of the outbreak. Specifically, we find a strong and significant correlation between travelling time and incidence during 2014 (R^2^=0.84; p-value<0.0001; **Fig. 1b**). However, during the second epidemic wave in 2015, the relationship between incidence and travelling time to the epidemic origin is considerably weaker and more diffuse (R^2^=0.1014; p-value=0.16; **Fig. 1c**). Further, the incidence time series for 2015 has shifted towards the annual pattern typically observed for DENV (**Fig. 1a**), which is driven primarily by seasonal cycles in vector abundance^21^. This supports the hypothesis that CHIKV has transitioned from epidemic establishment caused by spatial dissemination from the location into which the virus was introduced to a pattern in which multiple neighbourhoods are now acting as secondary foci of transmission.

**Dynamics of Chikungunya in Feira de Santana, Bahia, Brazil, in 2014-2015:**
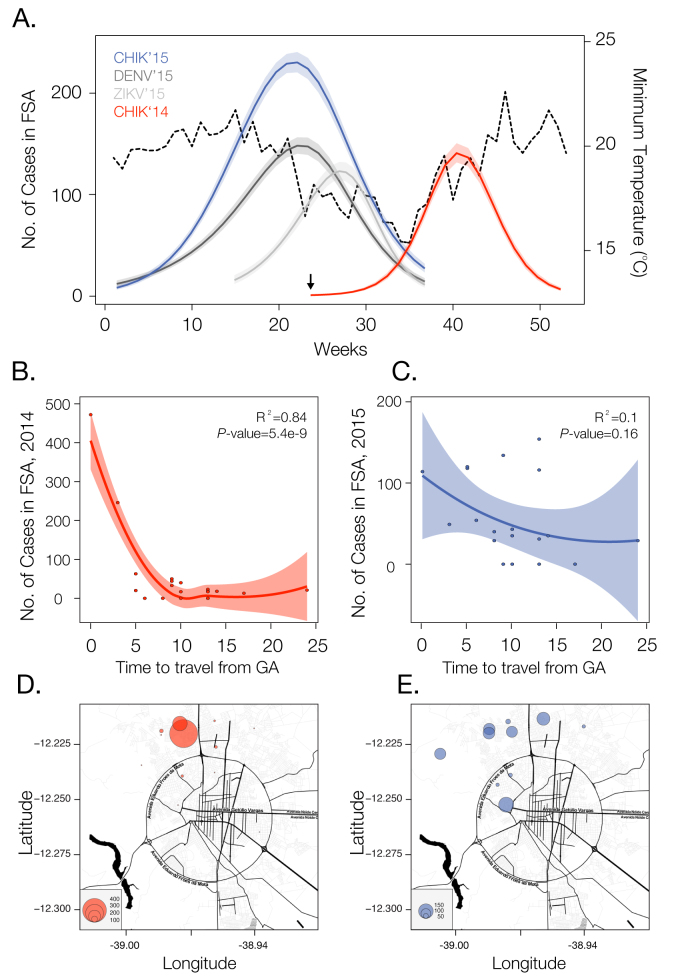
Incidence of CHIKV, DENV and ZIKV (number of clinically confirmed cases) in Feira de Santana (FSA) district, Bahia state, Brazil. The red curve shows CHIKV incidence between weeks 13 and 53 in 2014 (an arrow indicates the arrival date of the index case to George Américo, GA, district)). The blue curve shows incidence between weeks 1 and 35 in 2015. For comparison, the numbers of DENV (dark grey) and ZIKV (light grey) cases in 2015 are shown^20^. Curve fitting to incidence time-series data was performed using Poisson-smoothing in R software^22^. Panel (b) shows the correlation (R^2^ and p-value) between the estimated travel time (minutes) from GA to the geographic centre of each other urban district of residence in FSA presenting more than 10 cases in either 2014 or 2015, against the 2015 CHIKV incidence for each district in FSA. Panel (c) shows the equivalent results for 2014. Only urban districts with >10 reported cases in either year were considered. A map of Feira de Santana is shown with the number of cases per district of residence for 2014 (d) and 2015 (e).

Current data gives no indication that an endemic cycle will not be repeated in future years, as it is for DENV. Even if after the second cycle of CHIKV in FSA transmission is halted, as in the 2005-2006 outbreak in La Reunion^23^, it is likely that the virus will continue to spread in geographic areas with immunologically naïve hosts and an abundance of *Ae. aegypti* and/or *Ae. albopictus*. Both species are widely present in Brazil^11^ and are suitable vectors for the ECSA and Asian genotypes^24^. Future CHIKV transmission cycles are expected to be in phase with dengue and ZIKV seasonal epidemics, peaking when temperature, precipitation and humidity are propitious for *Aedes*-mediated transmission^25^. In addition, there is the potential for CHIKV to become endemic in the region, with occasional epidemics in humans. There is some evidence that CHIKV maintains an enzootic cycle in Southeast Asia involving nonhuman primates and forest-dwelling mosquitoes^26^, similar to the transmission of yellow fever virus in the Amazon and Orinoco river basin 27.

Since the establishment of CHIKV in FSA, the city has been experiencing a near-collapse of the public health system. The number of reported cases has already surpassed 5,363 but the local health care officials suspect that only 20% of cases are being notified. Although no treatment is available, several compounds and vaccines are currently under development^28^. More generally, the establishment of the ECSA genotype in Brazil is an unprecedented event in the Americas. This lineage is known to have acquired several adaptive mutations to *Aedes albopictus*. For example, a single amino acid mutation in its glycoprotein E1 (A226V) has been shown to confer 50-100 fold increases in transmissibility in this species^29^. Continued epidemiological and genetic surveillance and classification of CHIKV genotypes in Brazil and elsewhere in the Americas will be essential to monitor virus diversity, the acquisition of mutations related to viral reproductive success and vector specificity. In addition, along with ongoing DENV transmission, ZIKV is also spreading rapidly throughout northern Brazil^19^^,^ 30, and 1,459 suspected ZIKV cases have been reported until the 23 November 2015 in FSA^17^. The patterns of ZIKV match relatively well the epidemic cycles of DENV and CHIKV in 2015 and molecular epidemiological analyses are confirm that the CHIKV-ECSA strain has been circulating in Bahia state during 2015 (unpublished findings). Notably, the seasonal 2015 patterns of the arboviruses appear synchronized, especially in terms of their annual demise. These patterns reflect natural temperature cycles (**Fig. 1a**), which severely affect entomological and viral factors. Particularly, vector capacity is reduced when the temperature drops below 20 degrees Celsius, and transmission is completely disrupted once the temperature reaches 15 degrees Celsius^21^^,^ 25.

Other outbreaks of infectious disease have benefited from the use of digital surveillance for enhanced detection and response. Human mobility underlies many phenomena, including the spread of infectious diseases^31^^,^ 32 and high-resolution datasets that represent regional human population density and global mosquito vector densities have recently become available^11^^,^ 33. We have shown here that even the simplest mobility measures may be highly informative of virus spread. Anonymous mobile phone data may also provide a valuable source of information about patterns of human travel and could contribute to better forecasting of disease spread through space and time. Prediction of the dynamics and future disease burden of CHIKV in Brazil would benefit from the inclusion of temperature and precipitation variables that affect vector distributions^11^^,^ 21 ^,^ 25. In addition, high-resolution maps of the *Aedes albopictus* vector could complement LIRAa (Levantamento Rápido do Indice de Infestação por *Aedes aegypti*, http://portalsaude.saude.gov.br/), which is currently focused on mapping the infestation by *Aedes aegypti*. Finally, we note that it is exceptional for CHIKV, DENV and ZIKV to be all simultaneously circulating in one single city with < 1 million people. Such a scenario calls for national and international efforts to understand drivers of transmission, curb arbovirus transmission, train public health professionals to provide health care for the infected and effectively control mosquito populations in this municipality and beyond.

## Competing Interests

We declare we have no conflict of interests.
